# Smeared Multiscale Finite Element Models for Mass Transport and Electrophysiology Coupled to Muscle Mechanics

**DOI:** 10.3389/fbioe.2019.00381

**Published:** 2019-12-10

**Authors:** Milos Kojic, Miljan Milosevic, Vladimir Simic, Bogdan Milicevic, Vladimir Geroski, Sara Nizzero, Arturas Ziemys, Nenad Filipovic, Mauro Ferrari

**Affiliations:** ^1^Department of Nanomedicine, Houston Methodist Research Institute, Houston, TX, United States; ^2^Bioengineering Research and Development Center BioIRC Kragujevac, Kragujevac, Serbia; ^3^Serbian Academy of Sciences and Arts, Belgrade, Serbia; ^4^Faculty of Information Technologies, Belgrade Metropolitan University, Belgrade, Serbia; ^5^Applied Physics Graduate Program, Rice University, Houston, TX, United States; ^6^Faculty for Engineering Sciences, University of Kragujevac, Kragujevac, Serbia

**Keywords:** mass transport, drug delivery, electrophysiology, muscle mechanics, tumor model, liver, composite smeared finite element

## Abstract

Mass transport represents the most fundamental process in living organisms. It includes delivery of nutrients, oxygen, drugs, and other substances from the vascular system to tissue and transport of waste and other products from cells back to vascular and lymphatic network and organs. Furthermore, movement is achieved by mechanical forces generated by muscles in coordination with the nervous system. The signals coming from the brain, which have the character of electrical waves, produce activation within muscle cells. Therefore, from a physics perspective, there exist a number of physical fields within the body, such as velocities of transport, pressures, concentrations of substances, and electrical potential, which is directly coupled to biochemical processes of transforming the chemical into mechanical energy and further internal forces for motion. The overall problems of mass transport and electrophysiology coupled to mechanics can be investigated theoretically by developing appropriate computational models. Due to the enormous complexity of the biological system, it would be almost impossible to establish a detailed computational model for the physical fields related to mass transport, electrophysiology, and coupled fields. To make computational models feasible for applications, we here summarize a concept of smeared physical fields, with coupling among them, and muscle mechanics, which includes dependence on the electrical potential. Accuracy of the smeared computational models, also with coupling to muscle mechanics, is illustrated with simple example, while their applicability is demonstrated on a liver model with tumors present. The last example shows that the introduced methodology is applicable to large biological systems.

## Introduction

Convection and diffusion are the fundamental processes regarding transport of molecules or particles (e.g., nanoparticles used in drug delivery) from the cardiovascular system to tissue and interior (cytosol and organelles) (Rushmer, 1976; Hall, 2016). The route of this mass transport goes from blood or lymph to (microenvironment), from extracellular space to intracellular space, and finally to organelles. Along that bidirectional path, molecules/particles pass through the well-known biological barriers: vessel walls and cell and organelle membranes.

The interdisciplinary scientific field where the mass transport is studied, related to drug delivery by applying nanoparticles as the drug carriers to tumor sites, is named oncophysics (Ferrari, 2010; Koay and Ferrari, 2014; Blanco et al., 2015). The fundamental physical principles that govern the mass transport rely on the pressure gradient in case of convection and on concentration gradient in case of diffusion. Of course, the physical process is affected by the physical and chemical characteristics of the transported molecules or particles and the surrounding medium (Reulen et al., 1977; Popel and Pittman, 1998).

Blood as the basic fluid carrying nutrients, oxygen, drugs, and so on is a complex medium, and in computational models, certain approximations have to be adopted. In large vessels, this approximation is made by modeling blood as a homogenous fluid with certain viscosity properties (Shi et al., 2011), where the molecular transport is governed by convective-diffusive laws. This approximation is less valid in small vessels, such as capillaries (diameter size on the order of 10 μm; Mathura et al., 2001) where the presence of cells, mainly red blood cells (RBCs), affects transport (D'Apolito et al., 2015); still, in a simplified analysis, blood may be considered as a homogenous fluid with appropriate viscosity (Sevick and Jain, 1989b; Cinar et al., 1999). Complexity of the capillary network can be seen in various references (e.g., Skinner et al., 1990; Ma and Zhang, 1998), and images are available today on the Internet.

Tissue is a complex medium through which the mass transport occurs, but, regarding the computational modeling, it has to be approximated; the degree of approximation goes from an isotropic continuum to detailed description of extracellular space, cells, and cell interior. Extensive experimental research has been carried out over decades; we cite few recent, as molecule transport within brain tissue (Nicholson, 2001) or (Swabb et al., 1974) where tissue glycosaminoglycan content and drug molecular weight were found to be important parameters determining whether extravascular transport is governed by diffusion or convection. Khaled and Vafai (2003) presented a review of models used for convection and diffusion, including heat transfer, whereas Nugent and Jain (1984) and Gerlowski and Jain (1986) studied diffusivity of dextran molecules in tumor interstitium.

Transport through biological barriers as vessel walls, or cell and organelle membranes, depends on the transport properties of these barriers, as hydraulic or diffusion coefficients, and on the size of the surface that separates the continuum domains. As an illustration, we cite here measurements of the volumes and cross-sectional areas of blood vessels of a dog (Rushmer, 1976), which show that the mass exchange occurs mostly at the capillary–tissue level. The capillary volumetric fraction (capillary density) is one of the important characteristics in the mass exchange (Kojic et al., 2017b), which will represent a basic parameter in our computational models.

As will be seen in the formulation of our computational models, we will rely on the usually adopted governing laws for transport within extracellular space: the Darcy velocity–pressure relationship for convection and Fick's law for diffusion (Kojic et al., 2008). Transport within tumors has additional complexities due to variability of vessel diameters and lengths (Roberts and Palade, 1997) and irregular blood vessel branching (Less et al., 1991), as well as due to geometric resistance (Sevick and Jain, 1989a) (a measure of network irregularities), viscous resistance (Sevick and Jain, 1989b), and RBC mechanical properties (Sevick and Jain, 1991). Jain (1988) summarized basic properties of blood flow within tumors, while data specifically related to transport through capillary walls are given in Jain (1987).

The fundamental process in living organisms represents mass exchange and transport within cells. Cell interior is very complex, composed of different compartments or entities, such as organelles, which are, together with the cell cytoskeleton, immersed into the cell fluid–cytosol (Keener and Sneyd, 2009). Mass transport within cells is a particular branch of biomedical research, and here we cite a few references addressing some aspects of mass transport and exchange. The factors that affect the transport within cells range from biochemical to mechanical to signaling pathways (Rangamani and Iyengar, 2007). Theoretical basis of biochemical processes within different intracellular compartments is formulated (Lauffenburger and Horwitz, 1996; Sun and Pang, 2008; Chu et al., 2013). A computational framework for modeling mass exchange within cells, formulated as a virtual cell, is presented (Schaff et al., 1997; Moraru et al., 2008), which was further used in many applications (Slepchenko et al., 2003).

In electrophysiology, the goal is to determine the electrophysiological properties of all compartments and signal propagation characteristics within the body. For example, in heart electrophysiology, the fundamental advancements were achieved by designing the so-called clamp experiment (Hodkin and Huxley, 1952) in order to determine characteristics of the membrane currents and constitutive relations for the conduction of currents. Following this breakthrough achievement, further important experiments were performed, with modifications and extension of the constitutive relations (Noble, 1962; Baer and Rinzel, 1991; Decker et al., 2009; O'Hara et al., 2011). Based on these experimental results, a large number of computational models related to electrical signal propagation, which includes enormous complexities, have been introduced. For example, model presented (Noble, 1962) includes the role of various transmitting molecules—currents carried by ions through membranes and composite media (Ijiri et al., 2008) include intricate geometry such as in the case of Purkinje network. Since heart is a representative organ with electrical signaling coupled to mechanics, we here refer to a few computational models particularly related to heart modeling and mainly based on the finite element method. A review of computational methods for heart physiology can be found (Clayton and Panfilov, 2008). Among models discussed (Clayton and Panfilov, 2008) is the one related to coupling the electrical field with the mechanical response (Rocha et al., 2011; Lafortune et al., 2012; Dal et al., 2013).

Finally, in this short overview of mass transport and electrophysiology fields of research in science, technology, and medicine, we note that variation of electrical potential of cell membranes triggers other vital processes within living cells. A typical example is that calcium waves within muscle cells are induced by changes of the membrane potential. Calcium concentration within cells is fundamental for muscle contraction, and various mechanical models for muscles based on the calcium concentration changes are developed (Hunter et al., 1998; Kim et al., 2010; Mijailovich et al., 2010; Lafortune et al., 2012; Santiago, 2018). The variation of electrical potential fields also affects transport of charged particles or drug molecules (Schaff et al., 1997; Trapp and Horobin, 2005), and this kind of mass transport is included in our models (Kojic et al., 2019).

Considering the complexity of computational modeling of physical fields, such as pressure distribution, concentration of various molecules, or field of electrical potential within a body, or even within an organ, it is desirable to have a computational methodology feasible for practical applications. In our previous research, we have introduced a smeared concept for mass transport in capillary system and tissue (Kojic et al., 2017a,b, 2018, 2019; Kojic, 2018; Milosevic et al., 2018a) and demonstrated its superiority with respect to traditional modeling methods.

In this study, we summarize our previously formulated smeared methodology for modeling mass transport in biological tissue and electrophysiology problems and extend it to coupling with muscle mechanics. The motivation for the development of the models, which are applicable to real biomedical problems, is briefly outlined in the text above.

The paper is organized as follows. In the next section, we first summarize the governing equations of the gradient-driven processes: fluid flow through porous media, convective–diffusive particulate transport (including ions) and electrostatics; and then introduce a finite element (FE) form for these governing equations. In Section Smeared Model for Field Problems, the smeared methodology in a general form is summarized (Kojic, 2018), followed by specific forms related the problems described in Section A Summary of the Fundamental Equations for Gradient-Driven Physical Processes and FE Formulation. In Section Coupling Electrophysiology and Muscle Mechanics, we give a short description of coupling the smeared model for electrical potential with muscle mechanics. In Section Representative Results, we present several examples to demonstrate the main features of the smeared concept, including its accuracy and applicability; and, in the final section, we give a short summary and concluding remarks.

## A Summary of the Fundamental Equations For Gradient-Driven Physical Processes and FE Formulation

In this section, we first summarize the gradient-driven problems related to mass transport in blood vessels and tissue and electrophysiology. Then, we present a finite element formulation for these partial differential equations.

### Fundamental Equations for the Gradient Driven Field Problems

#### Flow Through Porous Media

In case of incompressible fluid flow through a porous rigid medium, the governing relation is represented by Darcy's law

(1) $$v_{i}=-k_{D_{ij}}\frac{\partial p}{\partial x_{j}},\;sum\;on\;j:\;j=1,\;2,\;3$$

where *v*_*i*_ is the Darcy velocity (as fluid flux per unit area of the continuum) in direction *x*_*i*_, *p* is fluid pressure, and *k*_*D*_*ij*__ is the Darcy tensor. The mass balance equation is

(2) $$k_{D_{ij}}\frac{\partial^{2}p}{\partial x_{i}\partial x_{j}}+q_{V}=0$$

WHERE *q*_*V*_ is a source term.

#### Diffusion

The constitutive law for diffusion is known as Fick's law

(3) $$Q_{i}=-D_{ij}\frac{\partial c}{\partial x_{j}}$$

and the mass balance equation is

(4) $$\begin{array}{l} -\frac{\partial c}{\partial t}-v_{i}\frac{\partial c}{\partial x_{i}}+\frac{\partial }{\partial x_{i}}\left(D_{ij}\frac{\partial c}{\partial x_{j}}\right)+q_{V}=0,\\ \;sum\;on\;i,j:\;i,j=1,\;2,\;3 \end{array}$$

Here, *c* is concentration, *Q*_*i*_ flux, and *D*_*ij*_ is the diffusion tensor. The generality is kept under the assumption that the diffusion tensor can be a function of concentration, that is, it can be *D*_*ij*_ = *D*_*ij*_*(c)*.

#### Electrostatics

The constitutive law is

(5) $$J_{i}=-G\frac{\partial V_{e}}{\partial x_{i}}$$

where *G* is electric conductivity, and *V*_*e*_ is electrical potential. The continuity equation for the current density can be derived from Maxwell equations in the form

(6) $$\frac{\partial }{\partial t}\left(\frac{\partial D_{i}}{\partial x_{i}}\right)=-\frac{\partial J_{i}}{\partial x_{i}},\;sum\;on\;i,\;i=1,\;2,\;3$$

where the current density components *D*_*i*_ can be related to the potential *V*_*e*_ as

(7) $$D_{i}=-\varepsilon \frac{\partial V_{e}}{\partial x_{i}}$$

where *ε* is the dielectric constant. Finally, the continuity equation is

(8) $$\varepsilon \frac{\partial }{\partial t}\frac{\partial^{2}V_{e}}{\partial x_{i}\partial x_{i}}=-G\frac{\partial^{2}V_{e}}{\partial x_{i}\partial x_{i}}+q_{V}^{e}$$

where $q_{V}^{e}$ is a volumetric source term (coming from ionic transport; Rangamani and Iyengar, 2007).

#### 1D Conditions

For further presentation, we give the expressions for the 1D conditions. For the fluid flow, the 1D conditions follow from the study of flow within pipes (Smith et al., 2002; Canic and Kim, 2003; Kojic et al., 2014). In case of a rigid pipe, the governing equation reduces to

(9) $$k_{pipe}\frac{\partial^{2}p}{\partial {\bar{x}}^{2}}=0$$

where $\bar{x}$ is the pipe direction, and *k*_*pipe*_ is the pipe coefficient, which can be derived from the so-called Hagen-Poiseuille law. Additional terms are present in the above equation for the case of deformable pipe (Kojic et al., 2014) but will not be considered in this work.

In case of diffusion, the 1D conditions follow from Equation (4). Hence, we have

(10) $$-\frac{\partial c}{\partial t}-v\frac{\partial c}{\partial \bar{x}}+\frac{\partial }{\partial \bar{x}}\left(D\frac{\partial c}{\partial \bar{x}}\right)+q_{V}=0$$

where $\bar{x}$ is the axis of propagation, and *D* is diffusion coefficient. In the case of electrical conduction, the governing equation has the form (9) with respect to the electric potential *V*_*e*_, where instead of *k*_*pipe*_, we have *G*_*a*_*A*, with A being the neural fiber cross-section.

#### Transport Through Membranes

Continuum domains of a composite media are often separated by membranes or walls in case of blood vessels or neural fibers. For the presentation of the smeared methodology, we here give the fundamental relations for transport through membranes. In case of fluid flow or diffusion, we have

(11) $$Q_{w}^{p}=k_{w}\left(p_{in}-p_{out}\right)$$

(12) $$Q_{w}^{c}=D_{w}\left(c_{in}-c_{out}\right)$$

with the flux of fluid $Q_{w}^{p}$ and mass due to diffusion $Q_{w}^{c}$ oriented outward (from *in* to *out*); *k*_*w*_ and *D*_*w*_ are wall hydraulic permeability and wall diffusivity, respectively. In the case of electrical field, the wall electrical flux relies on the so-called cable theory (Winslow, 1992). The outlet electrical flux (current density) *I*_*m*_ can be expressed as

(13) $$I_{m}=G_{m}\left(V_{e}^{in}-V_{e}^{out}\right)+C_{m}\left(\frac{\partial V_{e}^{in}}{\partial t}-\frac{\partial V_{e}^{out}}{\partial t}\right)$$

where *G*_*m*_ is membrane conductivity, and *C*_*m*_ is specific membrane (wall) capacitance.

### Finite Element Formulation

The above governing equations can be transformed into the FE equations of balance for a single finite element by implementing a standard Galerkin weighting method (Kojic et al., 2008). The incremental-iterative form of balance for a time step Δ*t* and iteration *i* can be derived in the form

(14) $$\begin{array}{l} {\left(\frac{1}{\Delta t}M+K^{v}+K\right)}^{\left(i-1\right)}\Delta \Phi^{\left(i\right)}=Q^{ext}+Q^{V}\\ \;-\frac{1}{\Delta t}M^{\left(i-1\right)}\left(\Phi^{\left(i-1\right)}-\Phi^{t}\right)-{\left(K+K^{v}\right)}^{\left(i-1\right)}\Phi^{\left(i-1\right)} \end{array}$$

where

(15) $$\begin{array}{l} M_{IJ}=\int_{V}c_{m}N_{I}N_{J}dV\\ K_{IJ}^{v}=\int_{V}v_{i}N_{I}N_{J,i}dV,\;sum\;on\;i:\;i=1,\;2,\;3\\ K_{IJ}=\int_{V}D_{km}N_{I,k}N_{J,m}dV,\;sum\;on\;k,m:\;k,m=1,\;2,\;3\\ Q_{I}^{V}=\int_{V}N_{I}q_{V}dV \end{array}$$

where **Φ** stands for pressure, concentration, or electrical potential as nodal variables; *N*_*I*_ are interpolation functions, *V* is element volume; *c*_*m*_ is mass coefficient (= 0 for fluid flow, and = 1 for diffusion); *D*_*km*_ for fluid is the Darcy tensor, while it is *G*δ_*i*__j_ (δ_*ij*_ is the Kronecker delta symbol) for electrical field. For the case of Darcy's flow or no convection, the convection matrix **K**^**v**^ is equal to zero. For electrical potential, we have that the “mass” matrix is

(16) $$M_{IJ}=\varepsilon \int_{V}N_{I,k}N_{J,k}dV,\;sum\;on\;k,\;k=1,2,3$$

and the convection matrix is equal to zero. Note that for 1D problems, the equations have the same form as the above, with one index *k* and no summation; and that the element volume is *V* = *AL*, where *A* is cross-sectional area and *L* is the element length. Note that Equation (14) assumes implicit integration scheme over time, that is, all variables are evaluated at the end of time step and at the current equilibrium iteration. This integration scheme is unconditionally stable and provides the best accuracy (Kojic and Bathe, 2005).

**Figure 1:**
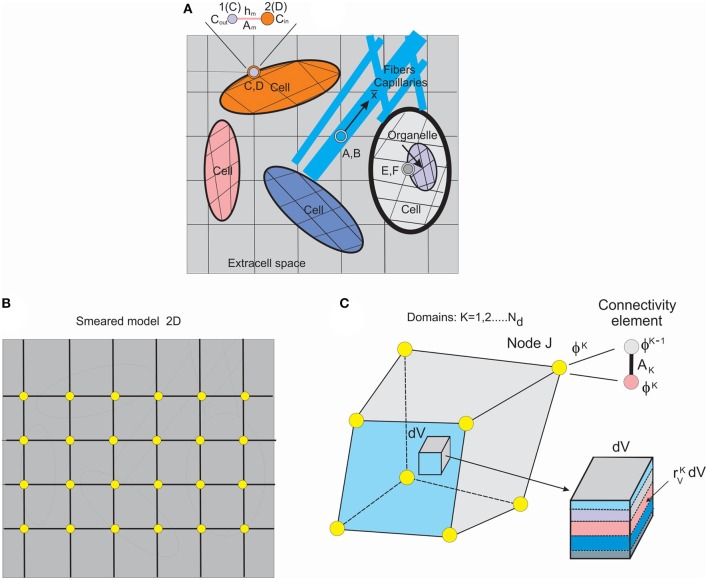
Schematic of detailed model and smeared model. **(A)** Detailed model of tissue as composite medium with continuum subdomains and capillaries/fibers, 2D representation, with continuum, 1D and connectivity elements. **(B)** Smeared finite element (FE) representation of the detailed model. **(C)** Composite smeared finite element (CSFE) with subdomains and connectivity element at a FE node J. (Used with permission from the *Journal of the Serbian Society for Computational Mechanics* from Kojic, 2018).

For modeling transport through the membranes (walls), we have introduced connectivity 2-node elements for nodes at membranes (Figure 1A) by using double nodes at the same space position at the membrane, with one node belonging to one side and the other to the other side of the boundary between two domains. The balance equation of the form (14) can be applied. The “mass” and transport matrices **M** and **K** can be written as

(17) $$\begin{array}{l} M_{11}=M_{22}=\frac{1}{3}c_{mm}A_{m}h_{m},\;M_{12}=M_{21}=\frac{1}{6}c_{mm}A_{m}h_{m}\\ K_{11}=K_{22}=-K_{12}=-K_{21}=D_{w}A_{m} \end{array}$$

where *A*_*m*_ is the area of the surface belonging to the node, *h*_*m*_ is the membrane thickness; in case of diffusion, *D*_*w*_ is the diffusion coefficient, while instead of *D*_*w*_, we have *k*_*w*_ and *G*_*m*_ for fluid flow and for electrical conduction, respectively; c_mm_ = 0 for fluid flow and *c*_*mm*_ = 1 for diffusion. For the case of electrical conduction, the non-zero terms of the “mass” matrix are

(18) $$M_{11}=M_{22}=A_{m}C_{m}$$

Finally, in this section, we summarize computational steps related to the incremental-iterative balance Equation (14). The steps are as follows:

1. Loop over time steps
  1.1 Loop over equilibrium iterations
    1.1.1 Loop over finite elements, evaluate, and store into global system element matrices and nodal vectors
    1.1.2 Solve for the nodal variables
    1.1.3 Check for the convergence criteria (unbalanced vector on the right-hand side are small and/or increments of nodal variables are small).
      If the convergence criteria are not satisfied, update the solution and go to next equilibrium iteration
    1.2.1 If convergence criteria are satisfied, store solution and go to the next time step 1.

END OF SOLUTION.

## Smeared Model for Field Problems

To introduce the smeared methodology (Kojic et al., 2014), we first consider a detailed model of a composite medium. A schematic of a medium composed of continuum domains-compartments together with a network of fiber-like 1D domain is shown in Figure 1A. The continuum domains include extracellular space, cells, and organelles. Capillaries and lymph vessels, and neural fibers, are represented by 1D elements within extracellular space, while cells can contain organelles—hence, the continuum domains have a hierarchical character. Each domain has its own FE mesh of continuum elements, while 1D domains have their own 1D finite elements with the coordinate axes along the elements ($\bar{x}$ axis depicted at one of these fibers).

Connectivity elements are shown as A, B; C, D; E, F; and enlarged at the top of the Figure 1A (the nodes C, D, also denoted as 1, 2). The two nodes, 1 and 2, have nodal values representing the two domains (ϕ_*out*_ and ϕ_*in*_ in the figure). The characteristics of the connectivity elements assigned to the boundary common nodes are membrane (or wall) transport coefficient, cross-section area *A*_*m*_, and the membrane (wall) thickness *h*_*m*_.

It can be seen from the detailed model description that significant effort is required to generate the model. The model generation would be an impractical or even impossible task in case of a complex medium such as tissue. In case of employing continuum FEs for membranes instead of connectivity elements, this task would be more demanding.

To overcome complexity issues, we further introduce a smeared model by formulating a continuum composite finite element (CSFE). The CSFE includes all constituents (continuum and 1D) in a way that the true physical fields, corresponding to a detailed model, are represented in a kind of average, so-called “smeared” sense, providing adequate accuracy. The smeared model for the same detailed model of Figure 1A, and with continuum elements only, is schematically shown in Figure 1B.

A few conceptual steps need to be considered to formulate the CSFE element. First, the 1D balance equations are necessary to be transformed into the corresponding continuum format. The derivations of the continuum transport tensor, for a general physical field, are given (Kojic, 2018) and can be expressed as

(19) $$D_{ij}=\frac{1}{A_{tot}}\sum_{K}D_{K}A_{K}\ell_{Ki}\ell_{Kj}$$

where *A*_*tot*_ is the total area of 1D domains in a reference volume; *A*_*K*_ is cross-sectional area of element K; *D*_*K*_ and ℓ_*Ki*_ are transport coefficients along the 1D elements and directional coefficients, respectively.

Each domain in the CSFE formulation has its own field within the corresponding volume of the CSFE. Therefore, as shown in Figure 1C, the FE node of the CSFE has a number of degrees of freedom ϕ^*K*^ equal to the number of domains *N*_*d*_. The domain volume *V*_*K*_ can be expressed as

(20) $$V_{K}=r_{V}^{K}V,\text{ and d}V_{K}=r_{V}^{K}dV$$

where $r_{V}^{K}$ is the volumetric fraction, and *V* is the total element volume.

Corresponding domains are coupled using connectivity elements placed at each node of the CSFE and in accordance to previously described connectivity elements in the detailed model. The cross-sectional area *A*_*JK*_ of a connectivity element at node J for the domain K can be expressed in the form

(21) $$A_{JK}={\left(r_{AV}^{K}V_{K}\right)}_{J}={\left(r_{AV}^{K}r_{V}^{K}V\right)}_{J}$$

where $r_{AV}^{K}$ is the area coefficient expressed as $r_{AV}^{K}=A_{K}/V_{K}$, and *V*_*J*_ is the volume of the total space of the continuum belonging to the node *J*. This volume can be evaluated as

(22) $$V_{J}=\sum_{el}\int_{V_{el}}N_{J}dV_{el}$$

where summation *el* goes over all finite elements with the common node *J*. Note that for convenience of modeling of any non-homogenous property of the material system, all of the surfaces, volumes, and volumetric and area ratios are assigned to FE nodes.

The finite element balance equations for continuum and connectivity elements are of the same form as in detailed model (Equation 14) with the matrices

(23) $$\begin{array}{l} M_{IJ}=\int_{V}c_{m}N_{I}N_{J}r_{V}^{K}dV\\ K_{IJ}=\int_{V}D_{ij}N_{I,i}N_{J,j}r_{V}^{K}dV,\;sum\;on\;i,j:\;i,j=1,\;2,\;3\\ Q_{I}^{V}=\int_{V}N_{I}q_{V}r_{V}^{K}dV \end{array}$$

where the material parameters are as in Equation (15). In case of electrical field, the matrix **M** in Equation (16) is now

(24) $$M_{IJ}=\varepsilon \int_{V}N_{I,k}N_{J,k}r_{V}^{K}dV,\;sum\;on\;k,\;k=1,2,3$$

and the source nodal vector due to ionic transport of a molecule *m* is (Kojic et al., 2019)

(25) $$Q_{I}^{mE}=\frac{DFz^{m}}{RT}\int_{V}N_{I}\frac{\partial }{\partial x_{i}}\left(c^{m}\frac{\partial V_{e}}{\partial x_{i}}\right)r_{V}^{K}dV$$

where *D* is diffusion coefficient, *z*^*m*^ is the molecule valence, *F* is the Faraday constant, *R* is the gas constant, *T* is absolute temperature, and *c*^*m*^ is concentration. The surface areas entering into matrices of the connectivity elements are as given in Equation (21).

The above concept has been implemented for diffusion and fluid transport through complex systems consisting of capillary network and tissue, with inclusion of the hydrophobicity effects in the connectivity elements (Kojic et al., 2014, 2017a, 2018), and with improvements of accuracy of the smeared methodology achieved by the correction function introduced in Milosevic et al. (2018a). The robustness and applicability of the smeared model are demonstrated (Milosevic et al., 2018b), where the CSFE is extended for modeling drug release from a complex mesh of drug-loaded nanofibers.

## Coupling Electrophysiology and Muscle Mechanics

Muscles (here assumed skeletal muscles) in the body are activated by electrical signals transmitted from the central nervous system to muscle cells. The signals trigger muscle activation since they produce a change in the cell membrane potentials, which further leads to flow through membrane of ions vital for cell functioning, such as potassium, sodium, calcium, and others (Hodkin and Huxley, 1952; Noble, 1962; O'Hara et al., 2011). The ion flow is bidirectional through various biological mechanisms. There are a number of mathematical models that connect the membrane potential change with activation of muscles. For example, for cardiac muscle, the mathematical expressions for generation of the so-called active stress along the muscle fiber, which produces the muscle contractile force, the membrane potential is used directly (Dal et al., 2013) or through the concentration of calcium *Ca*^2+^ within the muscle cell (Hunter et al., 1998; Lafortune et al., 2012; Berberoglu et al., 2014). The calcium *Ca*^2+^ is the crucial molecule that catalyzes the biochemical cycle of conformational change of muscle fiber molecules and therefore transformation of chemical into mechanical energy. Hence, in modeling muscle mechanical action, it is necessary for these models to have the calcium concentration change within muscle cells over time. We will use a widely accepted relation (Hunter et al., 1998)

(26) $$\sigma_{act}=\frac{{\left[Ca^{2+}\right]}^{n}}{{\left[Ca^{2+}\right]}^{n}+C_{50}^{n}}\sigma_{\max}\left[1+\eta \left(\lambda -1\right)\right]$$

where σ_*act*_ is the active stress along the fiber, *Ca*^2+^is calcium concentration, σ_max_is maximum isometric stress, *C*_50_ is concentration for 50% availability of actin sites for the crossbridge binding, *n* is related to the rate of this availability to concentration, η is parameter that is governing the rate of muscle fiber deformation, and λ is the fiber stretch.

Computational models of skeletal muscles and their implementation to the physiological conditions have been the subject of intensive research within the computational community (e.g., Kojic et al., 1998; Stojanovic et al., 2007; Mijailovich et al., 2010, and references therein).

We use velocity formulation, that is, the nodal variables are velocities—convenient to couple solid and fluid mechanics (Isailovic et al., 2014; Kojić et al., 2015), while stresses in solids are calculated from strains or stretches. The balance equation of a finite element can be written in the form (Kojic et al., 2008)

(27) $$\left(\frac{1}{\Delta t}M+\Delta tK\right)\Delta V^{\left(i\right)}=F^{ext}-F^{\mathrm{int}\left(i-1\right)}-\frac{1}{\Delta t}M\left(V^{\left(i-1\right)}-V^{t}\right)$$

where the mass and stiffness matrices **M** and **K** have a standard form (Kojic et al., 2008), and **V** and **V**^**t**^ are nodal velocities at the current (or previous) iteration and at start of time step, respectively; **F**^ext^ and **F**^**int**^ are external and internal nodal forces; nodal variables are one-dimensional arrays. Specific to muscle deformation is that, besides the material stress dependent on the state of deformation, there exists the active stressσ_*act*_ entering into the internal force vector as noted above.

## Representative Results

We have selected two numerical examples to illustrate the main characteristics of our smeared modeling methodology. The first example shows applicability of smeared models to large biological systems; here, a liver with two tumors is modeled. In the second example, accuracy of solutions is investigated on a small sample of the heart wall tissue, including coupling between electrophysiology and mechanics. The models are built into our FE code PAK (Kojic et al., 2010) with the corresponding CAD interface developed at R&D Center for Bioengineering BIOIRC.

We prepared animations for both examples, which are given in Supplementary Material.

### Liver Model With Tumors—Application of the CSFE

**Figure 2:**
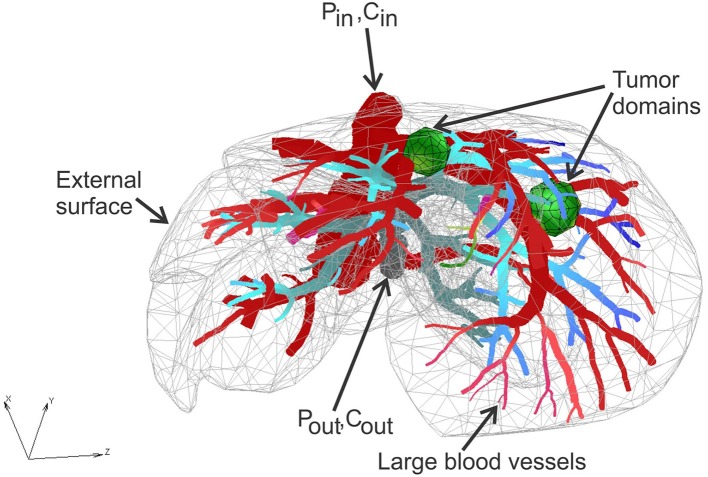
Liver model: geometry, tumor domains, and pressures within large vessels.

Liver model is based on geometry used (Kojic et al., 2017b), with two additional spheres modeled to mimic two tumor domains. Micro computed tomography (micro-CT) was used by the Preclinical Imaging Core at the Houston Methodist Research Institute to scan the vascular structure of a mouse liver. The geometry of the liver and blood vessel network is generated from micro-CT scan using procedure presented (Zagorchev et al., 2010). The geometry of the model is shown in Figure 2.

The FE model consists of 39,832 3D CSFEs, 7,736 1D pipe FEs for larger vessels, 726 connectivity elements for connecting large vessels with continuum nodes of smeared FEs. Two separate tumors within liver are with a total of 316 3D FE elements. The total number of nodes is 54,590. Data for this example are the same as in Kojic et al. (2017b).

Prescribed conditions in larger vessels (at input/output nodes of 1D pipe elements mesh) are: Inlet Pressure is 3,999.7 Pa (30 mmHg), Outlet Pressure is 1,333.2 Pa (10 mmHg), Inlet Concentration is bolus type c(t), presented in **Figure 6**, and outlet concentration is set to be 0.

Characteristics of fluid/diffusion flow through blood vessels (large vessels and capillaries) are: viscosity is 10^−3^ Pa s, and diffusion coefficient is 1,000 mm^2^/s.

Characteristics of blood vessel walls are: hydraulic permeability coefficient is 10^−12^ mm/(Pa s), diffusion coefficient is 0.1 mm^2^/s, and thickness is 10% of the vessel diameter.

Characteristics of tissue are: diffusion coefficient is 0.1 mm^2^/s, and Darcy coefficient is 10^−12^ mm^2^/(Pa s).

Parameters of smeared model are: average capillary diameter is 0.025 mm, capillary wall thickness is 0.0025 mm, volume fraction is 10%, diffusion coefficient of capillary wall is 10^−6^ mm^2^/s, and hydraulic permeability of capillary wall is 10^−12^ mm/(Pa s). The adopted values of material and geometric parameters are according to literature data, for example, Rushmer (1976), Gerlowski and Jain (1986), Jain (1988), Keener and Sneyd (2009).

Time steps used in simulations are: 40 × 2.5 s.

Additionally, there are two tumors in the model with the following characteristics:

**Table:** 

	**Tumor 1**	**Tumor 2**
Diff. coeff in extracellular space [mm^2^/s]	100	10
Darcy coefficient [mm^2^/(Pa s)]	1	0.1
Hydraulic coefficient [mm/(Pa s)]	1	0.1
Diffusion coefficient in small		
capillaries [mm^2^/s]	100	10
Diff. coeff of capillary wall [mm^2^/s]	100	10
Partitioning of capillary wall	0.8	0.7
Diff. coeff within cells [mm^2^/s]	100	100
Diff. coeff of cell membrane [mm^2^/s]	100	100

As can be seen from these data, we assumed 10 times smaller diffusion coefficient within tumor 2 than in tumor 1 to show the difference in concentration between these tumors.

With these material data and boundary conditions, we have solved for pressures and concentrations within the liver and two tumors using our smeared methodology. Some of the results are summarized below.

**Figure 3:**
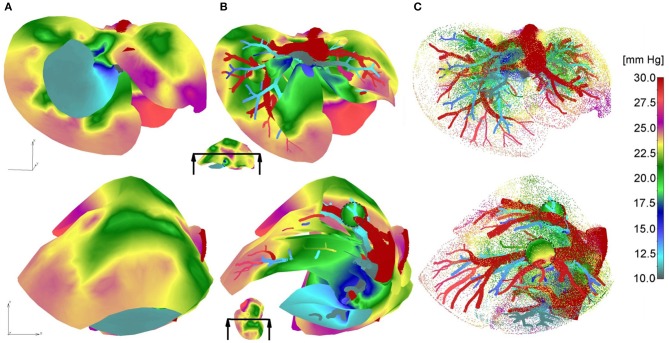
Pressure fields for two views: **(A)** full mesh; **(B)** clipped mesh; **(C)** dotted representation of results in tissue, and with full mesh in tumors.

Pressure fields for two views of the model are shown in Figure 3 for the outer surface of 3D smeared elements, cross-section, and a dotted representation of large vessels and continuum. Tumor surfaces are indicated by dashed lines. As can be seen from this figure, there is evident reduction in pressures, starting from large vessels to capillaries and further to tumors and healthy tissue.

**Figure 4:**
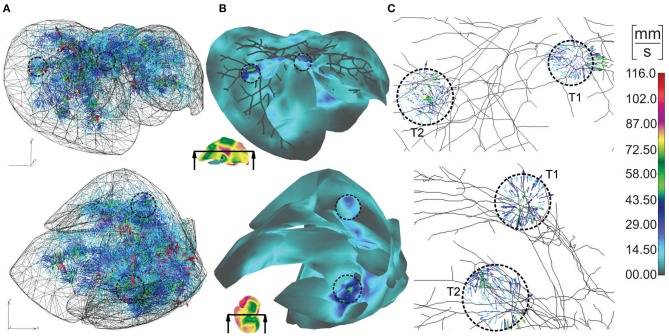
Velocities within liver model for two views: **(A)** vectors of velocities; **(B)** outer surface with clipped mesh; **(C)** vectors of velocity within tumors (enlarged images).

Vectors of velocities, field of velocity on the outer surface with a clipping plane, and vectors of velocities within tumors, for two different views on the model, are shown in Figure 4. For one of the tumors, T1, there is a positive pressure difference with respect to the surrounding tissue, which induces velocity vectors pointing out of tumor, that is, fluid is flowing out of tumor (Figure 4C–T1).

**Figure 5:**
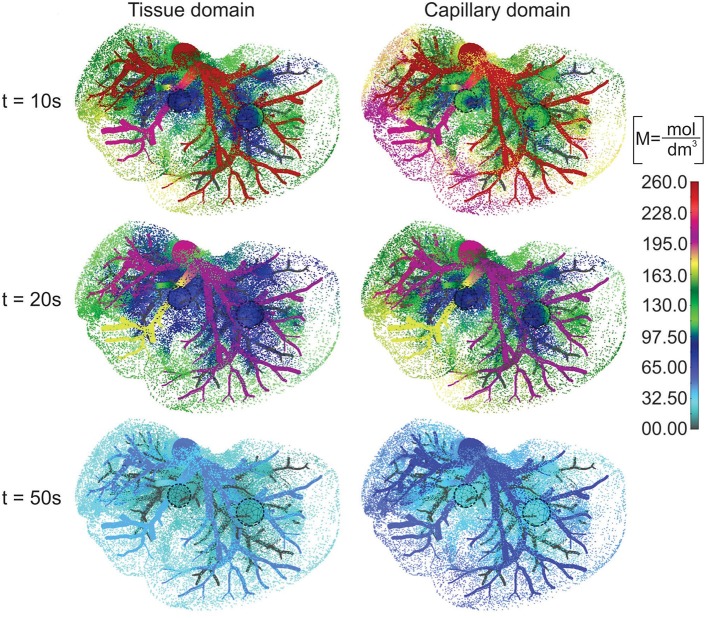
Concentration field in liver with tumors (marked with dashed lines), dotted results in tissue domain and with full mesh in tumors, for times *t* = 10, 20, and 50 s.

The concentration field within large vessels, liver tissue, and tumors is shown in Figure 5 for three time points. The largest values of the concentrations are noticeable in blood vessels, following a decrease going to capillaries and tissue. Also, concentration within tumor T2 is smaller compared to that of tumor T1 due to reduced diffusion coefficients.

**Figure 6:**
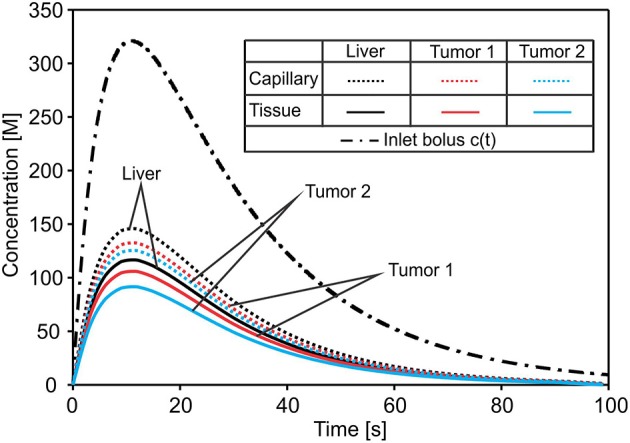
Concentration evolution in liver. The inlet concentration c(t) at large vessel has a bolus character and generates bolus-type profiles of mean concentrations in capillaries and tissue of the liver and within tumors—reduced with respect to c(t). The lowest concentration is in tumor T2 with the smallest diffusion and partitioning coefficient.

Change of the mean concentration within capillaries, tissue of liver, and tumors T1 and T2, is shown in Figure 6. In the first period of the process of drug transport within the liver, concentration within capillaries, tissue, and tumors is increasing according to increase of the entering mass (concentration). The concentration within capillaries reaches maximum with respect to the maximum of the entering c(t), which is also the case for tissue and tumor domains. It is evident that concentration in tumor T1 is higher than in tumor T2 due to the larger diffusion and partitioning coefficients.

### Electrophysiological and Mechanical Model of the Heart Wall

**Figure 7:**
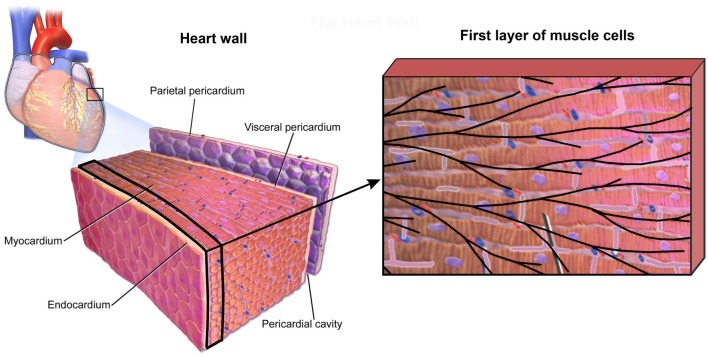
Small domain of heart wall tissue according to Blausen Medical (2014) **(Left panel)** and first layer of muscle cells close to sub-endocardium with mesh of Purkinje fibers projected on it **(Right panel)**.

To investigate accuracy of our CSFE model, a small sample of heart wall tissue is selected (Figure 7) following data (Blausen Medical, 2014; Santiago, 2018). From this model, we extract the first layer of muscle cells in myocardium, which is close to the sub-endocardium: the domain that includes the Purkinje fibers.

**Figure 8:**
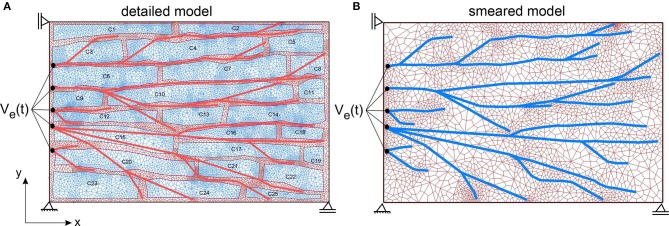
**(A)** The detailed heart wall model with cells and a network of Purkinje fibers. **(B)** Smeared model with tissue and Purkinje fibers associated to nodes of the CSFEs in a smeared manner.

According to the image in Figure 7 (right panel), the detailed 2D model is generated (Figure 8A), which consists of the mesh of 1D Purkinje fibers and 25 cells. Dimension of the model is 230 ×150 μm, volume fraction of cells is *r*_V_ = 0.71, and area/volume ratio of cell is *r*_AV_ = 0.18. Based on the detailed model, we generated the smeared model (Figure 8B) for calculation of the electrical potential and calcium current and concentration (O'Hara et al., 2011). FE nodes of 1D Purkinje fibers are connected with 2D FE nodes of extracellular space domain using connective 1D elements. As shown in Figure 8, it is assumed that the left vertical boundary of the tissue is constrained to displacements in *x* direction, and lower horizontal boundary is constrained to displacements in *y* direction. We assumed that muscle fibers have longitudinal direction with respect to the muscle cells, that is, they are aligned to the *x* direction.

Data used in the model are: electric conductance (*G*_*i*_, *I* = *x,y,z*) of extracellular space (further called tissue), cells, and in neural fibers is 1,000 [AV^−1^m^−1^]; specific membrane conductance (*C*_*m*_) of Purkinje fibers is 1,000 [S/μm^2^]; specific membrane capacitance of Purkinje fiber's membrane and cell membrane is 1,000 [AsV^−1^μm^−2^], and *G*_*m*_ = 0 for the fiber membrane. Diffusion coefficient of Ca^2+^ for tissue and cell is assumed to be 1,000 [μm^2^/s], while it is assumed that there is no diffusive transport through cell membrane. Material parameters of the muscle mechanical model (Equation 28), used in this example, are: *n* = 0.4, $C_{50}^{n}=0.5$, η = 0.2, and σ_max_ = 100kPa.

**Figure 9:**
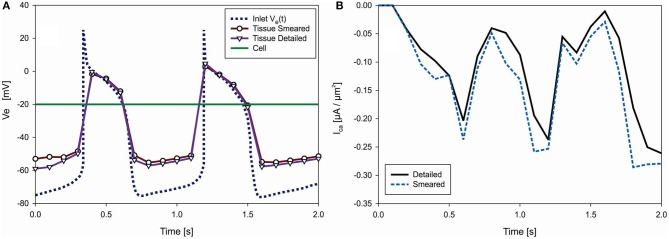
**(A)** Change of electric potential over time in extracellular space (tissue) domain—detailed and smeared model, with prescribed V_e_(t) at inlet nodes of Purkinje fibers and prescribed V_e_ = −20 V within cells (green). **(B)** Change of mean current density I_ca_ [μA/μm^2^], which affects transport of Ca^2+^ through cell membrane, according to detailed and smeared models.

The function of the electric potential is taken from Noble (1962): it consists of two identical cycles and is prescribed at inlet nodes of the Purkinje mesh [*V*_e_(t) in Figure 8]. We assumed constant potential inside cells (*V*_e_ = −20 mV). Accumulated current density (I_ORd_) in cell membrane is calculated according to ORd model (O'Hara et al., 2011) and added to Equation (13) of the FE solution procedure. For these conditions, change of the mean electric potential within tissue is shown in Figure 9A. Results are almost identical for both detailed and smeared models. Currents of ORd model that affect concentration of the Ca^2+^ in myoplasmic compartment are: *I*_*pCa*_,*I*_*Cab*_, and *I*_*NaCa, i*_; while currents that affect concentration of *Ca*^2+^ in the subspace compartment are *I*_*CaL*_ and *I*_*NaCa, ss*_. Mean current density *I*_*Ca*_ for transport of the *Ca*^2+^ ions can be calculated as (O'Hara et al., 2011):

(28) $$\begin{array}{l} I_{Ca}=-\left(I_{pCa}+I_{Cab}-2\;\cdot \;I_{NaCa,i}\right)\frac{V_{myo}}{V_{myo}+V_{ss}}\\ \;-\left(I_{CaL}-2\;\cdot \;I_{NaCa,ss}\right)\frac{V_{ss}}{V_{myo}+V_{ss}} \end{array}$$

where *V*_*myo*_ and *V*_*ss*_ are volumes of the myoplasmic compartment and subspace compartment, respectively. Change with time of the mean current density obtained by using detailed and smeared model is shown in Figure 9B, where I_ca_ for the detailed model is calculated as average over all cells.

**Figure 10:**
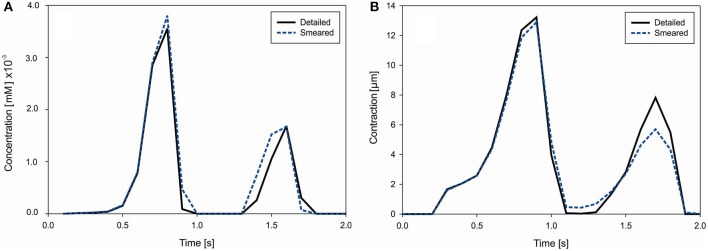
**(A)** Concentration change of Ca^2+^ in cells due to cell membrane currents. **(B)** Mean contraction (displacement) of the right vertical boundary of heart tissue segment due to Ca^2+^ change in muscle cells.

Mean concentration in cells, ${\left[Ca^{2+}\right]}_{mean}$, is calculated as average concentration in cells composed of myoplasmic (denoted by index “*i*”), subspace (“*ss*”), network SR (“*nsr*”), and junctional SR (“*jsr*”) compartments, according to O'Hara et al. (2011)

(29) $$\begin{array}{l} {\left[Ca^{2+}\right]}_{mean}=({\left[Ca^{2+}\right]}_{i}\;\cdot \;V_{myo}+{\left[Ca^{2+}\right]}_{ss}\;\cdot \;V_{ss}\\ \;+{\left[Ca^{2+}\right]}_{nsr}\;\cdot \;V_{nsr}+{\left[Ca^{2+}\right]}_{jsr}\;\cdot \;V_{jsr})/V_{cell} \end{array}$$

where *V*_*myo*_ = 0.68*V*_*cell*_, *V*_*ss*_ = 0.02*V*_*cell*_, *V*_*nsr*_ = 0.0552*V*_*cell*_, and *V*_*jsr*_ = 0.048*V*_*cell*_. Concentrations of Ca^2+^ in each compartment (*i, ss, nsr* and *jsr*) of ORd model are calculated according to equations provided in Supplementary of Reference (O'Hara et al., 2011). Change of the mean concentration within cells (cell domain), for both detailed and smeared models, is shown in Figure 10A.

Muscle contraction occurs from the generation of active stress according to Equation (26), where concentration of calcium is evaluated by our transport models (detailed and smeared). The mechanical response is calculated using the equation of motion (27). Mean contraction (displacement) of the right vertical tissue boundary is shown in Figure 10B. The largest contractions occur at *t* = 0.9 s and 1.6 s, which are in accordance with the Ca^2+^ concentration within cells.

**Figure 11:**
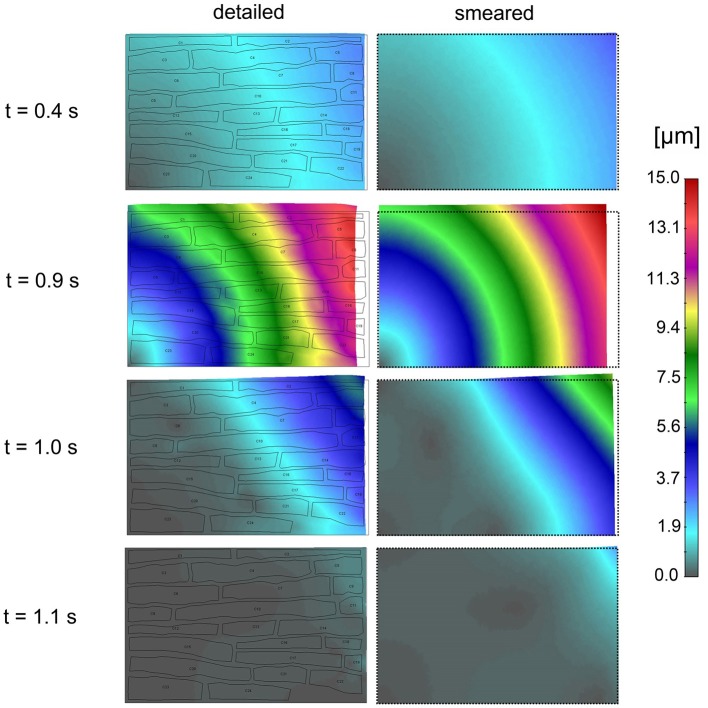
Effective contractions (displacements) according to the detailed model **(Left panel)** and smeared model **(Right panel)** for four time points (0.4, 0.9, 1.0, and 1.1 s) of first cycle of action potential function (inlet *V*_e_ (t) in Figure 10A).

Effective contraction (modulus of the displacement vector) field of the tissue for the first cycle of action potential function *V*_e_(t) is shown in Figure 11. It can be seen that the largest contraction occurs at *t* = 0.9 s. Good agreement is observed between the results of the two models.

**Figure 12:**
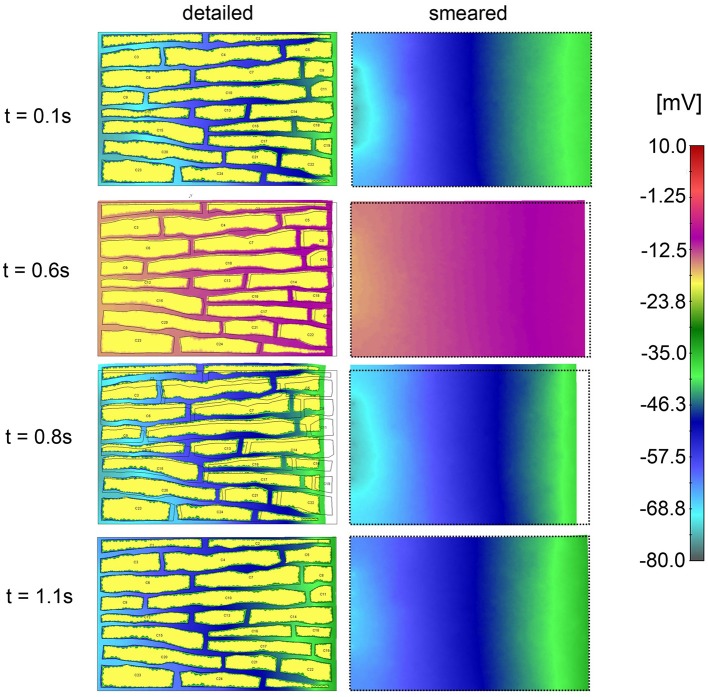
Electric potential according to detailed model **(Left panel)** and extracellular space of smeared model **(Right panel)** for four time moments (0.4, 0.9, 1.0, and 1.1 s) of the first cycle of action potential function.

Electric field potentials within extracellular space for four time points, according to detailed and smeared model, are shown in Figure 12. Potential within cells is kept constant *V*_e_ = −20 mV. Agreement between solutions of the two models is noted.

## Discussion

The main aims of the study were first to demonstrate applicability of our previously published general smeared finite element formulation for the physical fields to large-scale biological problems and second to include electromechanical coupling as the fundamental process for the muscle activation.

Considering the first aim, we note that, by using the today available computational models, software, and hardware, it is practically impossible to calculate drug distribution within an entire organ with different scales, from macro- to nano-, and modeling in detail blood vessels, cells, extracellular space, and even cell interior. On the contrary, our smeared FE methodology offers a platform to achieve solutions for this multiscale problem. We have selected for demonstration a liver of a mouse and generated continuum model with the geometry obtained from images. The model is simple since it consists of 3D continuum finite elements, with the 1D FEs for larger blood vessels as the only addition. The model, for the sake of generality, includes two tumors with different transport properties. The transport parameters data, including hydraulic and diffusive components, are taken according to available references (Gerlowski and Jain, 1986; Sevick and Jain, 1989b, 1991; Less et al., 1991; Mathura et al., 2001; Hall, 2016). The results displayed here show the concentration field, pressure distribution, and fluid velocities in different domains that are quantitatively correct. Accuracy of the solutions can be examined by, first of all, comparison with experiments. Comparison with other available numerical solutions in literature, such as those related to one domain only (e.g., tumor), would be possible with imposing the appropriate boundary conditions in our smeared model. This is not done here, since accuracy of our smeared models was examined in detail in our previous references (Kojic et al., 2017a,b, 2018, 2019; Milosevic et al., 2018b).

Detailed comparison of the smeared models in electrophysiology with the traditional models is given (Kojic et al., 2019). It was found in that analysis that the smeared model is advantageous with respect to the traditional monodomain and bidomain models (e.g., Roth and Wikswo, 1986; Henriquez, 1993; Henriquez et al., 1996; Keener and Panfilov, 1996) (reviewed in Clayton and Panfilov, 2008; Clayton et al., 2011). The defficiency of the traditional models also is that they do not include volumetric fractions of the distinct domains, which obviously must affect the solution. On the other hand, our smeared model relies on the multiple continuum domains coupled by the appropriate connectivity elements. Furthermore, smeared models are simple for application as, for example, in case of the heart: instead to model in detail neural fibers, the entire His-Purkinje system of the heart (Vigmond and Stuyvers, 2016) can be modeled by using the conductivity tensor of the form (19) within continuum 3D finite elements.

With the field of electrical potential within Purkinje network, extracellular and cell interior domain, coupled by the connectivity elements, it is possible to calculate membrane potential that governs the change of the calcium concentration within muscle cells according to Equation (29). It is further straightforward to compute the active stress within muscle cells by employing Equation (26), as one of the commonly used relationships.

## Concluding Remarks

The presented smeared finite element formulation offers a platform for multiscale modeling of physical field within a complex medium. Being simple for application, it is suitable for modeling large biological systems, such as liver with tumors used as an example in this study. It is straightforward to implement this approach to other organs, as lung, heart, pancreas, and so on and even brain. The brain has additional complexities related to signal transmission, but the presented model of the Purkinje network and electrical field within tissue shows that a brain model can be developed. The demonstrated electromechanical coupling demonstrates that this methodology can be implemented to muscle modeling in general.

## Data Availability Statement

All datasets generated for this study are included in the article/Supplementary Material.

## Author Contributions

MK formulated the methodology. MM designed the examples and analyzed results. VS, BM, and VG performed the numerical simulations and prepared results. SN, AZ, NF, and MF provided the data and images for reconstructing the liver and electrophysiology examples.

### Conflict of Interest

The authors declare that the research was conducted in the absence of any commercial or financial relationships that could be construed as a potential conflict of interest.
