# Supplementary material for: Smeared Multiscale Finite Element Models for Mass Transport and Electrophysiology Coupled to Muscle Mechanics
Source: Front Bioeng Biotechnol. 2019 Dec 10;7:381. doi: 10.3389/fbioe.2019.00381 (PMC6914730; doi:10.3389/fbioe.2019.00381)
Supplement: Supplementary file 16 [file Data_Sheet_1.pdf]

Supplementary Material

2D Heart model:

Animation 1. Field of displacements in detailed 2D heart tissue model, accompanied by diagrams of mean potential and calcium concentration within cells. Potential and concentration follow the inlet potential within neural fibers.

Animation 2. Field of potential in extracellular space of detailed 2D heart model, accompanied by diagrams of displacements in X- direction and concentration of calcium.

Animation 3. Field of concentration in extracellular space of detailed 2D heart model accompanied by diagrams of displacements in X- direction and potential in tissue.

Animation 4. Field of potential in extracellular space of smeared 2D heart model, accompanied by diagrams of displacements in X- direction and calcium distribution.

Animation 5. Field of concentrations in cell of smeared 2D heart model, accompanied by diagrams of potential and displacements in X- direction.

Animation 6. Nodal velocity field (vector representation) of detailed 2D heart model, accompanied by diagrams of potential and displacements in X- direction.

Animation 7. Nodal velocity field of detailed 2D heart model, accompanied by diagrams of potential and concentration of calcium.

Animation 8. Nodal velocity field (vector representation) of smeared 2D heart model, accompanied by diagrams of potential and displacements in X- direction.

Animation 9. Nodal velocity field of detailed 2D heart model, accompanied by diagrams of potential and concentration of calcium.

Animation 10. Field of displacements of smeared 2D heart model, accompanied by diagrams of potential in extracellular space and displacements in X- direction.

Liver model:

Animation 11. Evolution of mean concentration field in tissue (represented by dots) of liver, accompanied by the mean concentration-time diagram. As it can be seen, concentration reaches maximum value after about 20s. The inlet concentration is a bolus- shape function.

Animation 12. Evolution of mean concentration field in capillaries (capillary domain), represented by dots, of liver accompanied by the mean concentration-time diagram. Concentration reaches maximum value after about 20s (larger than in tissue due to partitioning at the capillary wall surface). The inlet concentration is a bolus- shape function.

Animation 13. Evolution of mean concentration field (zoomed) in tissue of tumors, accompanied by the mean concentration-time diagram. Concentration reaches maximum value after about 20s. The inlet concentration is a bolus- shape function.

Animation 14. Evolution of mean concentration field (zoomed) in capillaries (capillary domain) of tumors, accompanied by the mean concentration-time diagram. Concentration reaches maximum value after about 20s. The inlet concentration is a bolus- shape function.

Animation 15. Pressure distribution in capillaries (dotted representation) of liver, , accompanied by the mean concentration-time diagram. Pressure reaches maximum value after about 20s. The inlet concentration is a bolus- shape function.
